# The Exposition in Its Medical Aspects

**Published:** 1901-09

**Authors:** 


**A Monthly Review of Medicine and Surgery.**

*EDITOR:*

WILLIAM WARREN POTTER, M. D.

All communications, whether of a literary or business nature, books for review and exchanges should be addressed to the editor: 284 FRANKLIN STREET, BUFFALO, N. Y.

VOL. XLI.—LVII. SEPTEMBER, 1901. NO. 2.

### The Exposition in its Medical Aspects.

THE Pan-American Exposition has been in operation for four months and now has become familiar to a vast number of people, either by personal visitation or through oral and written accounts of what has been seen and described by visitors.

That the verdict of this vast throng of witnesses is one of approval may be observed in the steadily increasing attendance, the totals, per diem, jumping from thousands numbered in the forties during the first week in August, to those bordering on the eighties in the last week of the scorpion month. This is a gratifying condition, but it should be increased to the hundred thousand a day mark for the next two months, and we have no doubt these figures will be reached.

To the medical man there is much of special interest to be observed and studied at the fair, in addition to the vast exhibition of art work, machinery, agricultural material, colonial specialties and products, government displays and so on, to a never-ending accumulation of everything that can be raised or made by the hand of man. In the August issue of the JOURNAL we published a lengthy review of the medical and surgical side of the Exposition, written by the special correspondent of the *Boston Medical and Surgical Journal*, who presented the subject in an attractive and readable form, displaying the trained observer in every paragraph.

We now present to our readers a similar review of the same field, written by the special correspondent of the *New York Medical Record*, who is an equally keen observer as well as writer.

There may be discovered some little repetition in these two accounts, but each presents his own ideas from separate viewpoints and, taken together, they will afford to physicians the best possible penpictures of the medical features of the exposition. We feel sure they will be read with interest, and we cannot urge the exposition too strongly upon the attention of all physicians who desire to be entertained, instructed, and amused during a pleasant vacation.

The *Medical Record's* article is taken from its issue of August 17, 1901, and is as follows:

NOTES ON SOME OF THE MEDICAL FEATURES OF THE PAN-AMERICAN EXPOSITION.

There is much in the Exposition at Buffalo to attract and hold the attention of scientific and medical men. A person desirous of viewing thoroughly and of studying with some care the numerous exhibits exemplifying progress in medicine and science, will not only need to have a considerable amount of time at his disposal, but will also find his task greatly facilitated if he has a knowledge of the location of the features of interest.

Upon entering the West Amherst Gate the first building that strikes the view is the Emergency Hospital, the general plan and functions of which were described at length in the *Medical Record* some three months' ago. It may nevertheless be mentioned that its province is wholly restricted to medical work, that serious cases are transferred to institutions outside the grounds, and that no patient is permitted to remain overnight. The building contains twenty-six beds, and is excellently equipped in accordance with the latest methods of sanitary science. The medical director of the exposition, Dr. Roswell Park, looks after its administration and with Dr. Vertner Kenerson, the physician directly in charge, visits the institution daily. The house staff is composed of six young medical men who take duty in turn, two at the same time. The nursing staff comprises four nurses. There is also in the service of the hospital an automobile ambulance in charge of medical students of the University of Buffalo.

The hospital during the three months of the Exposition's existence has treated upwards of 3,000 cases, the majority of which have been medical. It is satisfactory to note that there have been but a few cases of a grave nature and that the occurrence of sunstroke and heat exhaustion has been remarkably rare. This fact when taking into consideration the prevalence of excessive heat all over the country at the end of June and at the beginning of July is decidedly noteworthy, and says volumes for the salubrity of Buffalo.

By far the larger number of those who have made use of the hospital up to the present time have been women, while among the employees and temporary residents of the Exposition the foreign element has supplied the bulk of the patients. The complaints have been characterised for the most part by their simple nature, and there has been no suspicion of anything resembling an epidemic. Probably the diarrhea and stomach disturbances to which the foreigners have shown themselves especially prone may be attributed to an unaccustomed mode of living, and perhaps to injudicious eating and drinking. On the whole, however, the health of the Exposition inhabitants has been highly satisfactory, as has been also the health of the visitors to the great fair.

Those who are responsible for the management and supervision of the Emergency Hospital must be commended for the admirable manner in which their duties have been carried out, and they can lay the flattering function to their hearts that their work has been well appreciated by the general public.

The incubation of infants seems, to use an every-day colloquial expression, to have "caught on" in more than one sense of the word. This method, of course, is largely resorted to for the rearing of weakly infants, and it also would appear to be equally popular from an exhibition standpoint.

In London some few years ago, an exhibition of infants in incubators was an adjunct to Barnum's show, and at that time the *Lancet* passed severe strictures upon the procedure, declaring the scheme was deserving of grave censure, and that it was neither fitting nor right that hapless babes should be placed on exhibition for the sake of gain. While one may agree with the remarks of our contemporary so far as this side of the question is concerned, it nevertheless cannot be denied that incubators have been the means of saving the life of many a prematurely born infant—whether for its weal or woe is altogether another matter. As, however, it seems that incubators have come to stay, the object should certainly be to provide the very best of the kind. The invention of and improvements made in the couveuse have been dealt with fully at different times in the *Medical Record*.

The incubators, twelve in number, are placed around a well-lighted room of which the walls and floors are impermeable. The incubators are glass-covered, and the infant reposes upon a mattress of woven wire, padded with soft material. A card is placed above each incubator, stating the date of the occupant's birth, it weight, and other details. A uniform temperature is assured in the incubators by means of a thermostat. The heat is derived from a metal boiler, which can be heated by a Bunsen burner or by an ordinary lamp. Ventilation is provided by the introduction of fresh air into the incubator from outside, the air being sterilised by filtration through an antiseptic fluid and then through cotton. The infants are taken out of the incubator every two hours and removed to a small room fitted as a nursery, there being fed by wet nurses and inspected as to their condition of cleanliness.

Nearly all the infants on exhibition are stated to be seven months' children. Whatever may be thought of the incubation method of preserving life, it must be confessed that the exhibition at Buffalo is excellently conducted, and is one of the most popular of the entire exposition. Indeed it is quite amusing to watch the crowds that frequent the baby show, which but goes to prove that after all a spectacle of human interest is most attractive to all classes.

On one side of the Emergency Hospital a "creche" has been established, the lack of such a necessary haven of rest for weary little ones having been greatly felt. Its institution was largely due to the eloquent plea on behalf of tired mothers and children set forth recently in the *Boston Medical and Surgical Journal.* The creche will be undoubtedly welcomed with lively feelings of gratitude, and will not only be highly appreciated by mothers of small families, but will be the means of bringing to the exposition many parents with their quivers full who would otherwise be compelled to forgo a visit.

A few words may be said in regard to the sanitation of the Exposition as a whole. The buildings are almost without exception well constructed, and due attention has been paid to their ventilation and hygienic qualities generally. There is, however, one conspicuous and even glaring exception to this satisfactory state of affairs, and this too in a building which contains one of the most attractive and popular displays on the grounds, and which is largely frequented by visitors to the exposition. The ventilation and sanitary arrangements of the art galleries, are, to put the case mildly, extremely defective. When the weather is warm, the atmosphere of the interior of the structure devoted to the fine arts is oppressive to a degree, and judging from the odor which commonly prevails, there must be something very wrong with the plan upon which the sanitation has been conceived. Even an enthusiast in art, and one who is carried away by his love for the beautiful, is nevertheless rarely so unconscious of sublunary matters as to be unwitting of heat and altogether careless of an offense to his olfactory organs; while to the ordinary individual the discomforts of the art building are apt to outweigh the pressure of gazing upon the really fine specimens of the artist's and sculptor's craft contained therein.

Perhaps the most instructive and interesting display in the entire Exposition—at any rate to the medical man—is that made by the medical department of the army. This exhibit, which is under the direction of Captain Edward L. Munson, of the United States Army Medical Department, is the largest and most complete of any in the exposition, and represents a model brigade field hospital, exemplifying its equipment and working down to the smallest detail. The only drawback to the unqualified success of this practical presentation of army medical methods is that the space allotted is decidedly limited in extent. So that, although the brigade field hospital is in itself illustrative of its object, the drill of the hospital Corps has to be considerably curtailed owing to inadequacy of area.

The tents constituting the brigade field hospital are pitched in close proximity to the main Government Buildings, and are arranged in form of a cross, the medical and surgical tents, mess kitchen, and office tents being placed within the arms of the cross, a position shown by experience to be the most convenient as well as calling for the least amount of time and energy. One hundred beds are provided in the hospital for a brigade numbering 5,375 war strength; a proportion of 2 per cent. sick can thus be cared for.

The tents used are those invented by Captain Munson, and it is claimed for them that their ventilation is effective, and that the temperature of the interior is, in tropical climates, materially lowered. The test to which these tents have been put in Cuba and the Philippines is stated to have justified the encomiums passed upon them. The exhibit at Buffalo is the first that has been publicly given of the improved methods now in vogue in the U. S. Army Medical Department. The revised system is the outcome of the careful and prolonged deliberations of a board of medical officers, who made a painstaking study of army medical arrangements of the principal countries of the world, by personal observation or by correspondence. Any features worthy of adoption were embodied into the American system, the weak spots were eliminated or improved upon, and the assertion is now confidently made that no nation of the world can equal or even approach the Army Medical Department of this country as regards its organisation and more especially its equipment. Economy of space and reduction of weight of material has been the watchword, and this result has been reached not only without detriment to the health and comfort of the soldier, but rather to his advantage. The lesson learned in the war with Spain has been fully utilised, and drugs in sufficient varieties and quantities to last a field brigade at war strength, under active service conditions for 90 days, are carried with the dispensary tents of the hospital. Space is lacking to describe at length the many ingenious devices resorted to, for the purpose of packing within a small compass, and thereby rendering transportation easier. It will suffice to point to the new model medical chest as a striking example. This chest, while weighing but eighty pounds, or thereabouts, contains drugs and medical supplies enough to last in the ordinary way 1,600 men for 90 days. Three field chests—medical and surgical and steriliser—form a regimental set, one of which can be carried by one man, two by two men on a litter, and three constitute a mule load. The same judgment has been displayed to render transportation as easy as possible in the mode of packing the hospital furniture. Each table in a tent can be quickly transformed into an operating table, and, with the chairs, can be taken to pieces and placed in a small chest. The bedding and other impediments of a hospital tent are contained in a canvas bag. In fact, a tyro even in army medical matters cannot fail to be impressed with the ingeniously simple devices, thought out and put into practice for the behoof of the soldier, and to enable a field officer to keep in touch with troops on the march, and thus permit the Army Medical Department to perform its duties efficiently. The army hospital corps give twice in the day, on a plot of ground adjoining the brigade field hospital, a smartly-executed exhibition of an emergency drill. The army medical exhibit is quite one of the most popular features of the exhibition, and is daily thronged by a crowd of the interested and curious.

In the main Government Building the U. S. Marine Hospital Service has a comparatively small but sufficiently complete exhibit, illustrating in an effective manner the methods and material of the service.

The exhibits are under the charge of Mr. Samuel W. Richardson, hospital steward, whose duties for many years have been rather of an executive nature than those appertaining to the rank of steward.

Models are shown of quarantine stations and detention camps; models of a section of a ward and of a marine-hospital operating room; a Kinyoun-Francis vacuum chamber as used at quarantine stations for disinfection of clothing; a complete *x*-ray outfit; a traveling laboratory. This latter is an eminently useful and, indeed, under certain circumstances, an essential part of the service's equipment. It may be easily understood that at times specimens for bacteriological investigation or for chemical analysis must be examined at once, as many would spoil during transportation to the hygienic laboratory at Washington. Therefore, a traveling laboratory, furnished for making immediate bacteriological examinations and chemical analyses, such as used by the Marine Hospital Service, may be said to be a necessary adjunct to its outfit.

Another feature of this exhibit which is deserving of special mention is the system of clinical reports devised by Surgeon-General Wyman. Clinical record sheets are issued for the use of the ward physician, upon which he writes in the usual way a history of the case, treatment, diet, etc. The point, however, to which it is wished to draw particular attention is that upon the discharge or death of the patient, these sheets, together with the temperature chart, are bound in a paper cover, upon the outside of which are inscribed the name, malady, and age of the patient with other facts bearing on the case. The reports, thus bound, are then arranged in cabinets, under the head of the disease from which the patient suffered, and, further, in another part of the cabinets, the case is tabulated under the name of the patient. The merits of this system are obvious, as prompt reference can be had to the history of any case. It would be superfluous here to touch upon the excellent work done in the past by the Marine Hospital Service. The exhibits at the Buffalo Exposition are, however, a remarkably convincing evidence that it is the object of the service to retain and add to a well-earned reputation, and with this end in view, to spare neither brains nor time in improving the organisation and equipment and in taking any steps which would seem to tend to the perfecting of the establishment.

